# Stable Nuclei of Nucleoprotein Filament and High ssDNA Binding Affinity Contribute to Enhanced RecA E38K Recombinase Activity

**DOI:** 10.1038/s41598-017-15088-z

**Published:** 2017-11-02

**Authors:** Chih-Hao Lu, Ting-Tzu Chang, Chia-Chuan Cho, Hui-Cin Lin, Hung-Wen Li

**Affiliations:** Department of Chemistry, National Taiwan University, Taipei, Taiwan

## Abstract

RecA plays central roles in the homologous recombination to repair double-stranded DNA break damage in *E*. *coli*. A previously identified *recA* strain surviving high doses of UV radiation includes a dominant RecA E38K mutation. Using single-molecule experiments, we showed that the RecA E38K variant protein assembles nucleoprotein filaments more rapidly than the wild-type RecA. We also used a single-molecule fluorescence resonance energy transfer (smFRET) experiment to compare the nucleation cluster dynamics of wild-type RecA and RecA E38K mutants on various short ssDNA substrates. At shorter ssDNA, nucleation clusters of RecA E38K form dynamically, while only few were seen in wild-type RecA. RecA E38K also forms stable nuclei by specifically lowering the dissociation rate constant, *k*_*d*_. These observations provide evidence that greater nuclei stability and higher ssDNA binding affinity contribute to the observed enhanced recombination activity of the RecA E38K mutant. Given that assembly of RecA nucleoprotein filaments is the first committed step in recombinational repair processes, enhancement at this step gives rise to a more efficient recombinase.

## Introduction

RecA is an essential protein for prokaryotic homologous recombinational (HR) DNA repair. When the replication fork encounters a template strand discontinuity, one arm of the fork can detach to form a double strand break (DSB). In the HR DNA repair, the broken end of the detached DNA arm is processed into a 3′ single-stranded DNA (ssDNA) overhang. RecA then assembles on this ssDNA overhang to form active presynaptic filaments available for homology searching and the catalysis of strand exchange to maintain genomic integrity^1,2^. RecA also acts as co-protease to trigger the cleavage of LexA repressor required for the induction of SOS response in the presence of extensive DNA damage^3,4^.

A few RecA-related strains were previously identified that altered the SOS induction activity of RecA and its accompanying mutagenesis^5–7^. The *recA730* strain, encoding a RecA E38K variant protein, improves cell survival at high UV doses^8,9^. RecA E38K also produces an increase in SOS mutagenesis by mediating the cleavage of the UmuD subunit of DNA polymerase V^5,9^. In addition, a *recA730* strain also possesses a greater capacity to promote genetic recombination^8^. Collecting all the observations, it was suggested that a RecA E38K point mutant can repair damaged DNA more efficiently than the wild-type (wt) RecA^10,11^.

DNA homologous recombination includes several key steps: recombinase nucleoprotein filament assembly, homology search, joint molecule formation and strand exchange. After DNA end resection, nucleoprotein filament assembly is the key regulatory step in DNA homologous recombination, and it consists of two phases: a rate-limited nucleation step and a fast extension step^12,13^. Several accessory proteins stimulate RecA activity in filament assembly. For example, translocating RecBCD stimulates RecA loading onto ssDNA^14^, and the RecF, RecO, and RecR proteins promote the nucleation of RecA on SSB-bound ssDNA^15–18^. RecA E38K displaces SSB much more efficiently than the wild-type RecA protein^10,11^, and nucleates onto ssDNA without the aid of the RecFOR proteins. *In vivo*, RecA E38K gives rise to a constitutive SOS response reaction^8,10,19^. Furthermore, *in vitro*, RecA E38K binds more readily to ssDNA containing secondary structure than does wtRecA^10,20^. Is it possible that the enhanced DNA recombinase activity of RecA E38K results from its ability to assemble nucleoprotein filaments more efficiently? Understanding the stimulating mechanism of this point mutant could allow us to design a highly efficient recombinase for future gene editing applications. To address the enhanced activity of RecA E38K, we utilized two single-molecule approaches: tethered particle motion (TPM) and single-molecule fluorescence resonance energy transfer (smFRET) to study mechanistic details of the RecA E38K presynaptic filament assembly process. We found that RecA E38K exhibits faster nucleation and extension kinetics than wtRecA using TPM assembly experiments. In addition, the E38K mutant is able to nucleate on ssDNA with a smaller nucleation cluster size and bind to ssDNA more tightly compared to wtRecA.

## Results

### RecA E38K promotes homologous recombination more efficiently than wild-type RecA due to faster presynaptic filament formation

In homologous recombinational DNA repair, presynaptic filament formation and strand exchange are two key regulatory steps. The overall homologous recombination efficiency could be enhanced if the rate of nucleoprotein filament formation or strand exchange rate or both are stimulated. Previous studies showed that wtRecA and RecA E38K mutants have similar strand exchange efficiency once nucleoprotein filaments were pre-assembled^20^. To confirm that, we utilized a previously developed TPM outgoing strand experiment^21^ to monitor the RecA-mediated DNA strand exchange in real-time at the single-molecule level. The outgoing strand experiment is initiated by the introduction of pre-mixed RecA-coated nucleoprotein filaments into reaction chambers containing surface-immobilized homologous duplex DNA substrates (Figure S1A). One strand of the surface-bound, hybrid DNA is displaced by the RecA-bound nucleoprotein filaments during successful strand exchange and is labeled with a streptavidin bead at its 5′-end. Therefore, bead disappearance signals the completion of the successful RecA-mediated strand exchange^21^. Strand exchange efficiency is defined as the percentage of bead disappearance events after nucleoprotein filament addition. No significant difference in strand exchange efficiencies between wtRecA and RecA E38K (shown in Figure S1B) was found, consistent with the previous data^11,20^. The wtRecA data and “No RecA” control^21^ were listed for easy comparison. We also carried out bulk strand exchange experiments to compare the strand exchange efficiencies between RecA E38K and wtRecA (Figure S1C). 40 bp 5′-Cy3-labeled homologous dsDNA was challenged with pre-formed, 80 mer RecA E38K or wtRecA nucleoprotein filament to undergo strand exchange reaction. The reaction mixtures were then resolved by native polyacrylamide gel electrophoresis (native PAGE) (Figure S1D). Strand exchange efficiency, which is characterized by the fraction of 40/80 hybrid DNA product formation, is plotted as a function of time (Figure S1E). Native PAGE result also shows that RecA E38K and wtRecA lack apparent discrepancy in strand exchange kinetics, which is consistent with previous publications^20^ and our TPM outgoing strand experiments. These results confirm that once nucleoprotein filaments are pre-formed, RecA E38K shows no apparent additional stimulation during the strand exchange step.

**Figure 1:**
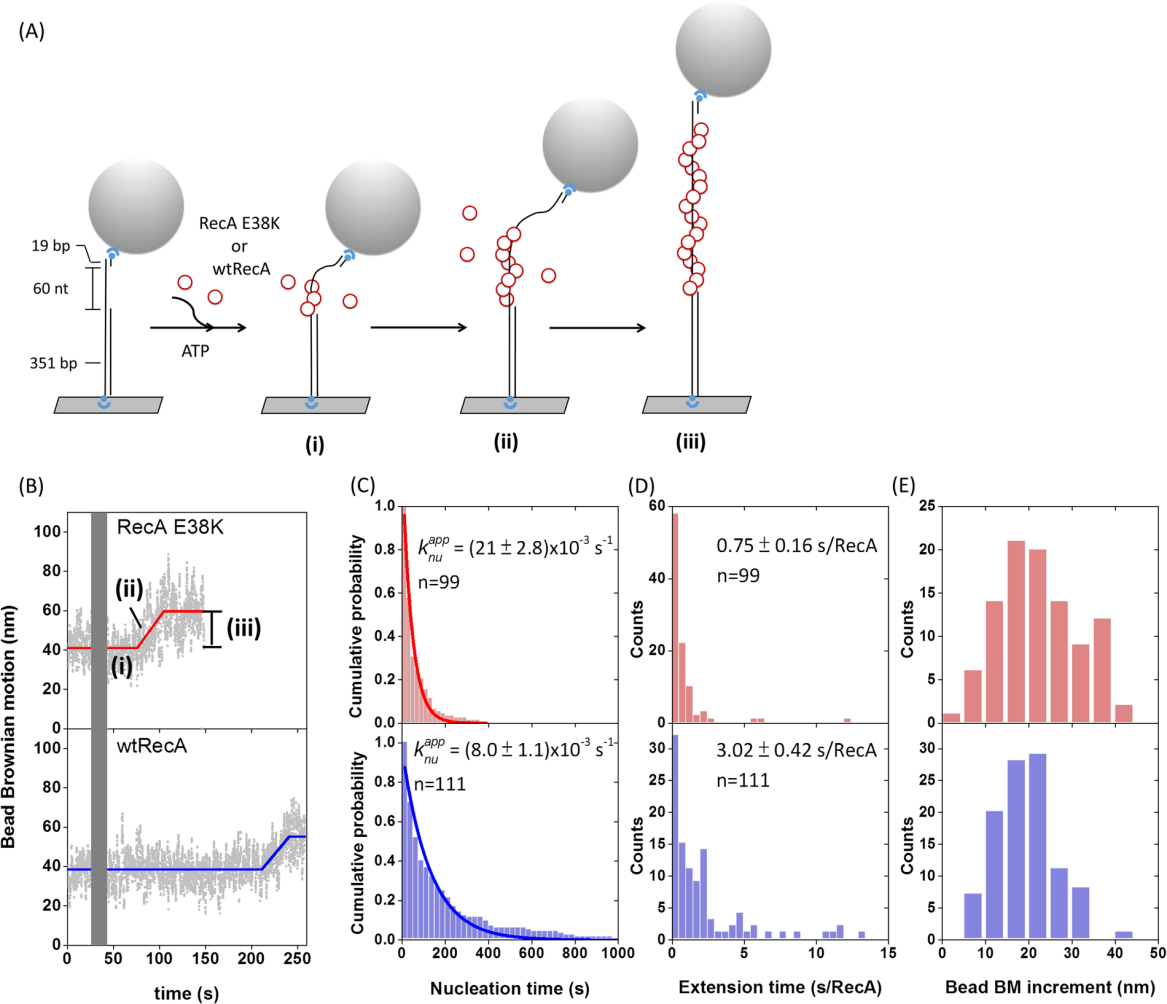
RecA E38K assembles into nucleoprotein filament faster than wild-type RecA. (**A**) Schematic illustration of TPM assembly experiment. Wild-type (wt) RecA or RecA E38K in the presence of ATP assembles onto the surface-anchored (dT)_60_ gapped DNA, leading to the increase in bead BM. (**B**) Representative bead Brownian motion time-courses of single-molecule assembly experiments using RecA E38K (upper) or wtRecA (lower). Gray bar represents the dead time during protein addition. (**C**) Nucleation time & rate (region (i) in Fig. 1B), (**D**) extension time (region (ii)) and (**E**) bead BM increment (region (iii)) of RecA E38K (upper) or wtRecA (lower). E38K mutant formed stable nuclei on (dT)_60_ ssDNA ~2.6-fold faster than wtRecA ((21 ± 2.8)x10^−3^ s^−1^ vs (8.0 ± 1.0)x10^−3^ s^−1^). Moreover, RecA E38K (0.75 ± 0.16 s/RecA) extended on ssDNA faster than wtRecA (3.02 ± 0.42 s/RecA) but they shared similar filament coverage on ssDNA. *n* indicates the number of tethers compiled from more than 3 independent experiments. Error bar of nucleation rate was calculated based on bootstrapping method; error bar of extension time is one standard error of mean.

Earlier biochemical studies showed that RecA E38K exhibits higher ssDNA-dependent ATPase activity than wtRecA at earlier times in the filament assembly process^11^, suggesting that E38K mutants could assemble more efficiently onto ssDNA. Here, we used the TPM experiments to compare the kinetics of the filament assembly process of the wtRecA and RecA E38K. The TPM assembly experiments were initiated by flowing either wtRecA or RecA E38K in the presence of ATP into the reaction chamber containing surface-bound (dT)_60_ gapped DNA (Fig. 1A). RecA assembly onto ssDNA leads to the DNA extension and an increase in BM^22^. In the reaction conditions used at room temperature, wtRecA or the RecA E38K mutant didn’t assemble onto the duplex DNA region over the experimental timescale, so any observed BM change resulted from the assembly on the ssDNA gap region^23^. The TPM assembly experiment enabled us to figure out the kinetic parameters during the assembly process, including the nucleation time ((i) in Fig. 1B), the extension time (defined as the time required to add RecA monomers to ssDNA) ((ii) in Fig. 1B) and the BM increment ((iii) in Fig. 1B)^22,24^. We defined the dwell time of DNA tethers between RecA addition and significant bead BM increase as nucleation time, which characterizes the time needed for RecA to form stable nuclei on ssDNA. After stable nuclei form, RecA rapidly extends on ssDNA to form long, stable nucleoprotein filaments and bring about significant bead BM increment. Extension time was calculated by dividing the duration time of continuous BM increase ((ii) in Fig. 1B) by total amounts of RecA subunits bound on ssDNA substrate at saturation (equivalent to one third of the number of nucleotides on ssDNA substrates). The conversion factor linking bead BM increment to the amounts of RecA subunits bound had been reported^24^. Exemplary time-courses of wtRecA and E38K nucleoprotein filament assembly are shown in Fig. 1B. Both assembly time-courses showed a rate-limiting step in nucleation of nucleoprotein filaments, followed by a fast extension step. RecA nucleation rates were calculated using maximum likelihood estimation (MSE) and bootstrapping method, which were utilized to measure protein-DNA interaction kinetics previously^25^. At the same recombinase concentration of 1 μM, the E38K mutant protein exhibited a much-enhanced rate of assembly: RecA E38K had a nucleation rate of (21 ± 2.8)x10^−3^ s^−1^ compared to the wtRecA nucleation time of (8.0 ± 1.0)x10^−3^ s^−1^. RecA E38K thus possesses a ~2.6-fold enhancement in the nucleation step under these conditions (Fig. 1C). Moreover, RecA E38K also extended on the ssDNA somewhat faster than wtRecA (Fig. 1D) but shared a similar degree of filament coverage with wtRecA (Fig. 1E). These observations indicate that RecA E38K forms nucleoprotein filaments much more efficiently than wtRecA *in vitro*, even in the absence of SSB. This is consistent with the enhanced ATPase rates associated with filament assembly observed previously for RecA E38K^10,11^. Combining results from single-molecule outgoing strand exchange and filament assembly experiments, our observations suggest that the molecular basis for more efficient RecA E38K mediated recombination *in vitro* is the faster nucleoprotein filament assembly rather than accelerating the strand exchange step.

### RecA E38K exhibits distinct nucleation dynamics on ssDNA compared to wtRecA, including a higher nuclei stability

**Figure 2:**
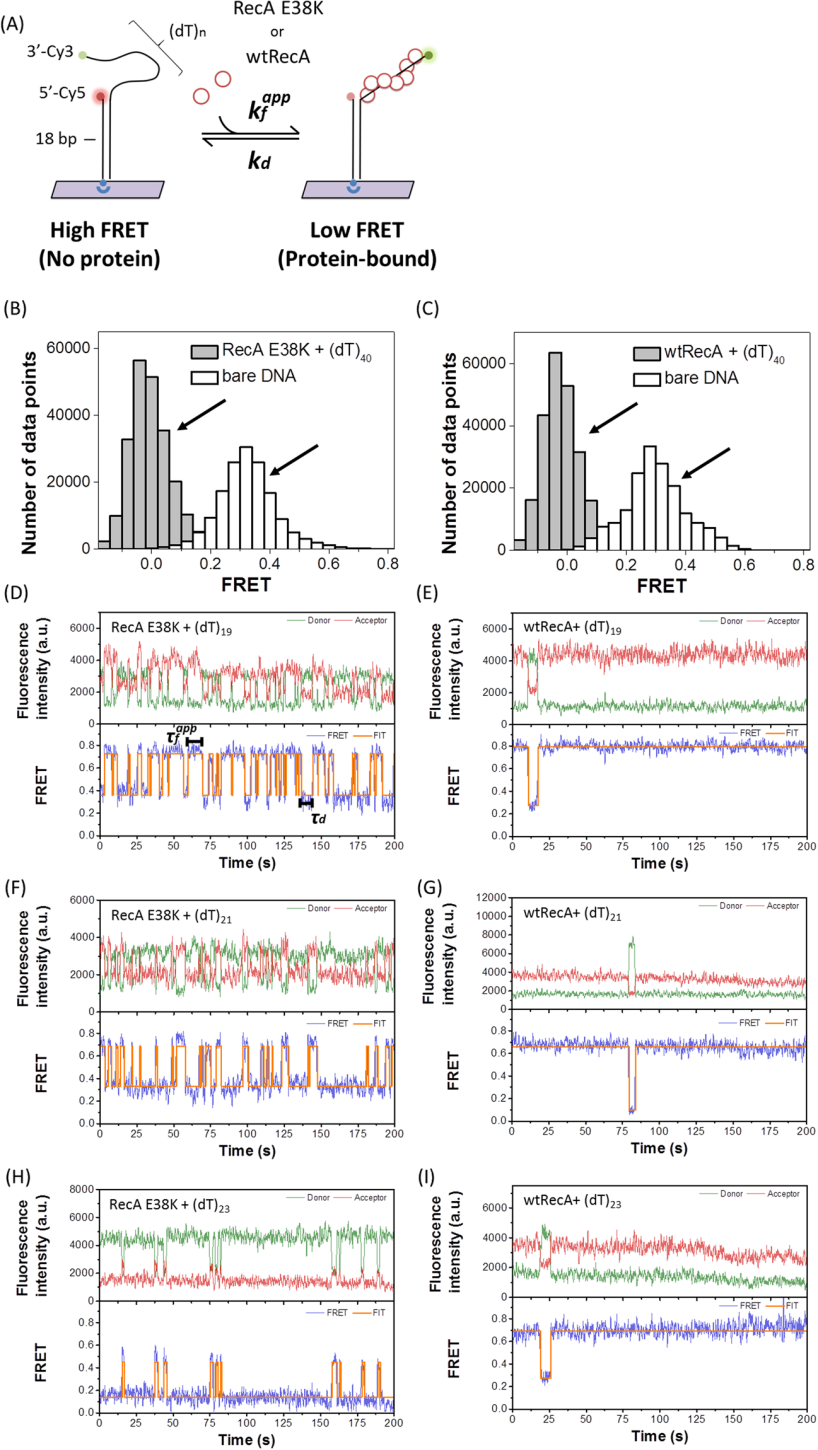
wtRecA and RecA E38K showed distinct nucleation cluster dynamics on ssDNA. (**A**) Schematic illustration of smFRET experimental setup. wtRecA or E38K mutant assembles onto ssDNA results in the FRET decrease due to the increase of dye pair separation. This experimental setup allows us to measure the apparent formation and dissociation rate constants of *k*_*f*_^*app*^ and *k*_*d*_ of wtRecA or RecA E38K nucleation cluster on individual DNA molecules. (**B**–**C**) FRET histograms of bare (dT)_40_ DNA substrates (empty bars, ~ 0.3 FRET value) shifted to zero as either RecA E38K or wtRecA were added. The nucleoprotein filaments were stable so no FRET alternation was seen. (**D**–**I**) FRET time traces of RecA E38K or wtRecA assembly at short ssDNA (dT)_n_ overhangs (n = 19, 21 and 23). Each time trace was fitted by the Hidden Markov model (orange line). FRET efficiency fluctuates between two states (high and low FRET). Most DNA molecules were at the low FRET state for RecA E38K, but were at the high FRET state for wtRecA among three DNA substrates, indicating that RecA E38K stays stably bound on these short ssDNA and wtRecA barely binds. All experiments were carried out in the presence of 2 mM ATP.

In TPM assembly experiment, we measured the nucleation time of RecA E38K and wtRecA. However, in this stage, bead BM did not change conspicuously although there are several binding and dissociation events of few RecA monomers during the nucleation process. To gain further insights into the RecA assembly dynamics, we examined the nucleation process with shorter ssDNAs. We took advantage of the single-molecule fluorescence resonance energy transfer method to see the nucleation dynamics of wtRecA or RecA E38K nucleation in high resolution. Our experimental design contains a 3′-biotinylated DNA strand annealed to a longer strand to generate 3′-ssDNA overhangs of various lengths (9, 15–23, 27, 33 and 40 nt), anchored on the surface of the streptavidin-coated, PEGylation-modified slide (Fig. 2A). The dye donor Cy3 and acceptor Cy5 placed at the 3′ ends of an overhang and the ss/ds junction, respectively, allow us to monitor the RecA binding onto the ssDNA region between the dye pair. In the absence of proteins, the ssDNA region is flexible, so energy transfer between donor and acceptor dye is efficient, leading to higher FRET signals (E ~ 0.6–0.9 for 9–27 nt substrates; E ~ 0.3–0.4 for 33 nt & 40 nt substrates, Fig. 2 and Supplemental Figure S3). Upon RecA binding to ssDNA, the separation between the dye pair increases, resulting in a lower FRET signal (E ~ 0.0–0.3, Fig. 2). This experimental setup allows us to measure the dwell times associated with the high-to-low FRET transition and the low-to-high FRET transition on individual DNA molecules, which in turn reflects the rate of RecA nucleation cluster formation and dissociation, respectively (Fig. 2A & D). RecA binding to the ssDNA should exhibit a length dependence on these short ssDNA substrates. We first used a long (dT)_40_ substrate where the ssDNA length is sufficient to form stable RecA nucleoprotein filaments (Fig. 2B–C & Figure S4B–C). As soon as wtRecA or RecA E38K were introduced, all FRET values were around zero. The high-to-low FRET transition took place within the flow deadtime, and no consequent low-to-high FRET transition was seen, confirming that both wtRecA and E38K form stable filament at this ssDNA lengths (binding fraction ~100%) and both proteins were active. Upon extensive buffer washing, RecA disassembled and the FRET value returned to ~0.3, excluding a potential dye photobleaching issue (Supplemental Figure S4D–S4E).

We then studied the nucleation cluster dynamics at short ssDNA lengths. For extremely short ssDNA lengths (<9 nt), no RecA binding occurs. For longer ssDNA overhangs, RecA binding remains unstable, so only transient binding events occur, resulting in fast alternation between a low FRET and a high FRET state in time-courses of both wtRecA and E38K, as seen in Fig. 2 and FRET histograms in Figure S6. For sufficiently long ssDNA lengths, the RecA nucleus becomes more stable, and a low FRET signal can be observed. It’s clear to see that the population of low-FRET molecules of both wtRecA and E38K increase with increasing ssDNA length (red peaks in Figure S6). When we compared the nucleation cluster dynamics of wtRecA and RecA E38K at various ssDNA lengths, we noticed two differences. First, E38K exhibited more rapid alternation dynamics compared to wtRecA ((dT)_19, 21, 23_ as examples shown in Fig. 2D–I) at the short ssDNA lengths. For example, E38K underwent multiple binding and unbinding cycles on (dT)_19_ substrates within 200 seconds (Fig. 2D); but binding events for wtRecA were much more infrequent (Fig. 2E). Second, the percentage of molecules showing FRET alternations was much higher for RecA E38K. As not all molecules with the intact dye pair showed examples of FRET alternation seen in Fig. 2D–I, some molecules didn’t have any change in FRET value within the 200-second observation window. We, therefore, defined the binding fraction as the percentage of molecules with FRET alternation within the observation time, reflecting the binding affinity for ssDNA of two proteins. As listed in Table 1, there exists a striking difference in the binding fraction between wtRecA and E38K. For example, for (dT)_19_ substrates, wtRecA interacted with only 3.0 ± 0.9% of DNA molecules, but 74 ± 8.1% of molecules were bound by RecA E38K and exhibited FRET alternation. The binding fraction of both wtRecA and RecA E38K are the function of ssDNA length, the binding fraction increases at longer ssDNA lengths. Note that binding fractions of both wtRecA and E38K are ~100% for (dT)_40_ substrates, excluding the contribution from inactive enzymes. Both the binding fraction and the FRET alternation frequency observations support the general idea that RecA E38K has a higher ssDNA binding affinity than the wtRecA. None of these reactions included SSB, and the ssDNA binding affinity is distinct from the enhancement in SSB displacement observed in earlier studies^10,11^.

**Table 1:** The mean binding fraction of and apparent nucleation frequency wtRecA and RecA E38K on the various ssDNA lengths. Binding fraction is defined as the ratio of numbers of DNA molecule undergoing assembling with RecA to total numbers of observed DNA molecules. Apparent nucleation frequency is defined as the numbers of assembly events per DNA molecule per hour. Error bar is one standard deviation from at least 3 independent experiments.

ssDNA length (nt)	Binding fraction (%)	Apparent Nucleation frequency (/hour/molecule)		
	wtRecA	RecA E38K	wtRecA	RecA E38K
9	N.D.	1.0 ± 0.7	N.D.	21.5 ± 5.74
15	N.D.	61 ± 6.7	N.D.	195 ± 63.5
16	N.D.	60 ± 7.9	N.D.	276 ± 41.0
17	N.D.	65 ± 6.6	N.D.	198 ± 26.1
18	N.D.	69 ± 16	N.D.	186 ± 11.5
19	3.0 ± 0.9	74 ± 8.1	0.63 ± 0.18	179 ± 92.8
20	N.D.	71 ± 8.6	N.D.	134 ± 56.7
21	3.9 ± 0.3	68 ± 11	1.15 ± 0.52	116 ± 55.5
22	N.D.	70 ± 4.9	N.D.	71.9 ± 14.0
23	7.3 ± 1.9	78 ± 8.6	2.10 ± 0.35	46.6 ± 14.9
27	13 ± 3.3	N.D.	3.45 ± 0.87	N.D.
33	99 ± 0.7	N.D.	1.33 ± 0.33	N.D.
40	~100	~100	~1	~1

**Figure 3:**
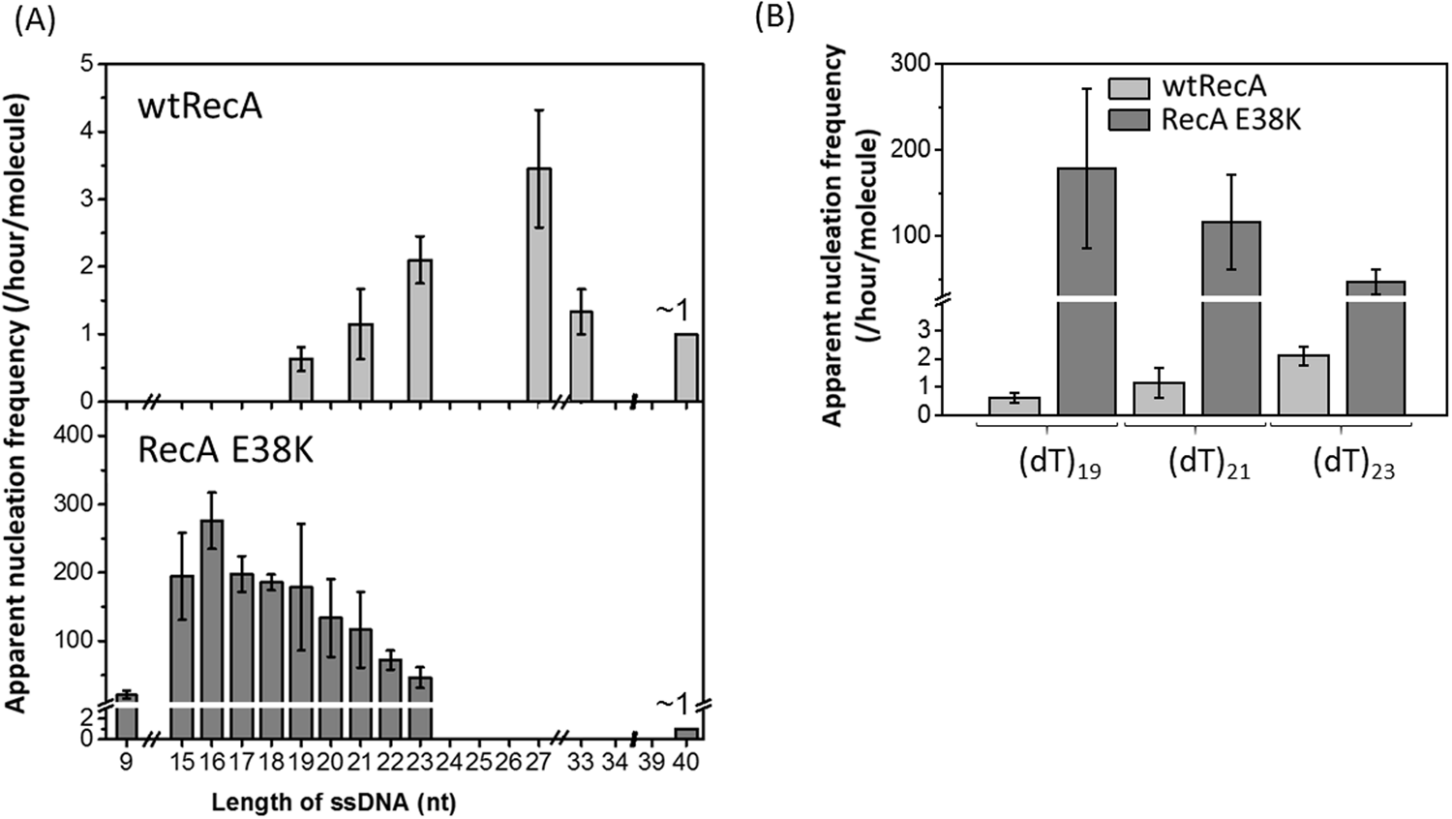
ssDNA length dependence on apparent nucleation frequency is different for wtRecA and RecA E38K. (**A**) The apparent nucleation frequency of wtRecA varies from 1–5 per molecule per hour and increases with ssDNA length from 19 nt to 27 nt and drops at 40 nt. The apparent nucleation frequency of RecA E38K is much higher, from 21.5 (per molecule per hour) at 9 nt ssDNA, to 276 at 16 nt ssDNA. It shows an initial increase at short ssDNA (9 nt) followed by a gradual decrease at longer ssDNA. (**B**) Comparison of apparent nucleation frequency of wtRecA and E38K at (dT)_19_, (dT)_21_ and (dT)_23_. Two orders of magnitude difference are seen. Error bar is one standard deviation from at least three independent experiments.

To quantitatively compare the nucleation events between wtRecA and RecA E38K, we defined the apparent nucleation frequency as the numbers of nucleation (binding) events of individual molecules within a given time interval. Each high-to-low FRET transition is regarded as one nucleation event. Numbers of nucleation events in each individual time-courses were counted within the 200 second observation time. Numbers of nucleation events in 200 seconds, multiplied by the binding fraction (percentage of molecules showing binding, Table 1), defines the apparent nucleation frequency, expressed in per molecule per hour for easier comparison (Fig. 3 and Table 1). When the ssDNA length is smaller than (dT)_19_, very few nucleation events were detected for wtRecA, suggesting that wtRecA requires a ssDNA longer than 19 nt to form a stable nucleation cluster. The apparent nucleation frequency for wtRecA gradually increased from (dT)_19_ as the ssDNA length increased (Fig. 3A). This observation could be interpreted by either (i) the stability of wtRecA nuclei on shorter ssDNA substrates (<19 nt) is very low, even wtRecA has the similar nucleation unit as E38K, or (ii) The ssDNA length is not sufficiently long for forming wtRecA nucleus. Previous studies suggested that ~ minimum 5–6 RecA monomers is required for a stable wtRecA nucleus^13,26^, so longer ssDNA (>18 nt) is required. In strong contrast, the apparent nucleation frequency of RecA E38K exhibited several big differences: (i) the nucleation frequency was 1–2 orders of magnitude higher than that of wtRecA at the short ssDNA lengths, and (ii) the apparent nucleation frequency was detected even with (dT)_9_, and increases rapidly with length until a gradual decrease was seen when ssDNA length was longer than 16 nt (Fig. 3A). For example, at (dT)_19_, the apparent nucleation frequency for wtRecA was 0.63 ± 0.18 per molecule per hour, but 179 ± 92.8 for RecA E38K. The nearly 100-fold difference in apparent nucleation frequency might be attributed to the difference in ssDNA binding affinity, consistent with the faster nucleation rate of RecA E38K seen in the assembly experiments (Fig. 1E). The ssDNA length dependence of RecA E38K’s apparent nucleation frequency shows a distinct distribution of initial increase and gradual decrease (Fig. 3B). As discussed in the wtRecA case, the increase in apparent nucleation frequency among various ssDNA length reflects the required ssDNA length hence the stability of RecA nucleation cluster increases with increasing ssDNA length. Sufficient ssDNA lengths are available for forming more stable nucleus and extending into a longer stable filament. The nucleation cluster dissociation events are then reduced at longer ssDNA lengths. Therefore, at longer ssDNA lengths, once nucleation event takes place, it tends to stay at the low FRET state. This leads to the decrease in apparent nucleation frequency seen for RecA E38K when the ssDNA length is longer than 16 nt (Fig. 3A). The apparent nucleation frequency of RecA E38K was detected at (dT)_9_ and the stability of RecA E38K nucleation cluster increases with increasing ssDNA length from 9 nt ssDNA. These suggest that the minimum size of nucleation cluster for RecA E38K is less than five E38K monomers, smaller than that for wtRecA. Our data can’t exclude the possibility that both wtRecA and E38K has similar nucleation cluster size, but with very different stabilities. When ssDNA length is sufficiently long, a stable RecA nucleus is formed and no dissociation event occurs. Thus, it is expected that the apparent nucleation frequency of wtRecA would drop once ssDNA becomes much longer (>27 nt, in Fig. 3A); For (dT)_33_ substrate, ~98% of wtRecA are stably bound to (dT)_33_ ssDNA tail without dissociating from ssDNA (Figure S5). For the (dT)_40_ substrate, the apparent nucleation frequency for both RecA E38K and wtRecA substrate is indeed dropped to ~1. The two-order-of-magnitude enhancement for RecA E38K apparent nucleation frequencies (Fig. 3B), as well as the higher stability of RecA E38K nuclei, contribute to the efficient nucleoprotein filament assembly for RecA E38K.

### Nucleation cluster formation and dissociation rate constants of E38K

**Figure 4:**
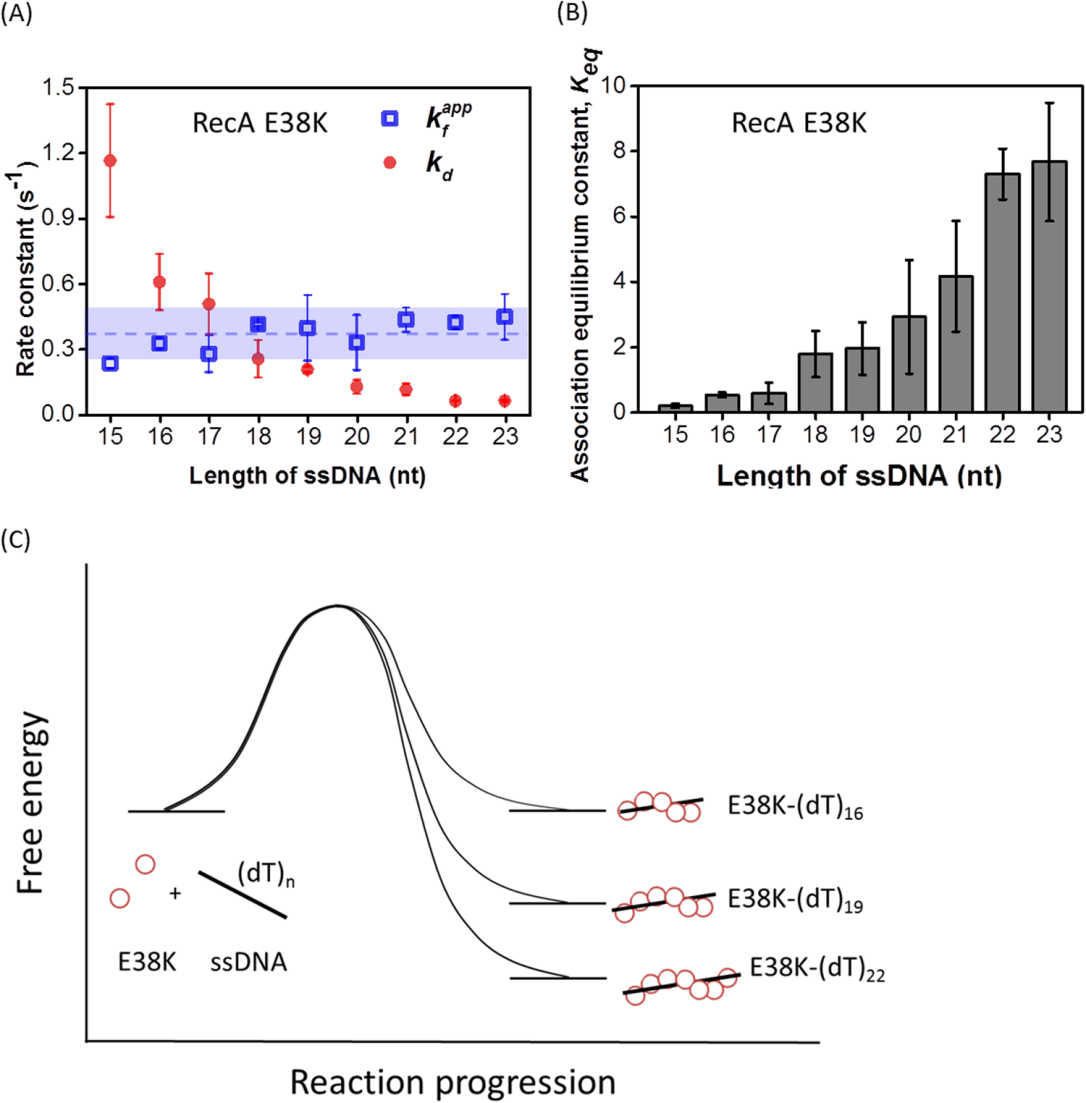
ssDNA length dependence on the rates of RecA E38K nucleation cluster formation and dissociation. (**A**) Apparent formation rate constants (*k*_*f*_^*app*^, blue open squares) of RecA E38K analyzed from the time-courses of Fig. 2 show no difference among 8 different ssDNA lengths (dashed line: mean of all *k*_*f*_^*app*^; blue shaded region: interval of one standard deviation of the mean). Dissociation rate constants (*k*_*d*_, red solid circles) decrease with increasing ssDNA lengths. (**B**) Equilibrium constants calculated based on data in (A), which is obtained by dividing *k*_*f*_^*app*^ to *k*_*d*_ of a given ssDNA length. Association equilibrium constants (*K*_*eq*_) increase at longer ssDNA lengths. (**C**) Energy diagram of E38K nucleating onto different ssDNA lengths. Error bar is one standard deviation from at least three independent experiments.

Nucleation cluster formation and dissociation events seen in the FRET time-courses of RecA E38K proteins (Fig. 2) allow us to determine the apparent formation and dissociation rate constants of nucleation clusters (*k*_*f*_^*app*^ and *k*_*d*_) during RecA E38K assembly. The fast alternation in FRET reflects the limited stability of the nucleation cluster on specific ssDNA lengths. Thus, the rates discussed here refer to the process before the stable nucleus is established. Once a stable nucleation cluster is formed, the RecA nucleoprotein filament can continue to extend and disassemble dynamically to be functionally active in HR. We analyzed the data for ssDNA lengths ranging from 15–23 nt for RecA E38K, since sufficient FRET alternations of these DNA substrates with RecA E38K makes the analysis possible. wtRecA, on the other hand, did not have sufficient DNA binding affinity at this ssDNA length range and exhibited only few alternations making analysis statistically not meaningful. The collected dwell times of the high-FRET (no protein) and the low-FRET (protein-bound) states can be both fitted to a single exponential decay (Fig. 2D–I and Supplemental Figure S7). Over the short ssDNA length used (15–23 nt), apparent rate constant for nucleation cluster formation, *k*_*f*_^*app*^, is nearly independent of ssDNA lengths (blue open squares, Fig. 4A) for RecA E38K. However, the rate constant for nucleation cluster dissociation, *k*_*d*_, diminishes as ssDNA length increases (red solid circles in Fig. 4A). This is expected as the nucleation process has the same transition state, so the *k*_*f*_^*app*^ is the same, as shown in the proposed energy diagram of Fig. 4C. Different *k*_*d*_ values at different ssDNA lengths at this length range imply the different stabilities of the assembled nucleation cluster states. Considering the increased protein-DNA interaction at larger nucleation cluster sizes, the decreasing *k*_*d*_ at longer ssDNA lengths reflects the higher stability. Association equilibrium constants, *K*_*eq*_ (ratio of rate constants) can then be directly assessed by the ratio of *k*_*f*_^*app*^ and *k*_*d*_ (Figure 4B)_._ Note that the ssDNA length-dependence of *K* comes from the reduction in *k*_*d*_ only since *k*_*f*_^*app*^ remains the same. We also figured out association equilibrium constant of RecA binding onto various ssDNA based on FRET histograms from Figure S6, which is equal to the ratio of low-FRET population (red peak) to high-FRET population (red peak). Association equilibrium constants acquired by two different approaches (Hidden Markov Model Fitting vs. initial experimental data inputs) are similar (Figure S8), confirming the reliability of the fitting.

## Discussion

Regulation of DNA homologous recombination is required for cell survival. Various accessory proteins participate in the HR process in order to activate or inhibit recombinase activity at the appropriate times. In *E*. *coli*, for example, RecFOR regulates HR by stimulating RecA loading on SSB-coated ssDNA^15,27^. A much more complex regulatory scheme exists in higher eukaryotes^28,29^. Understanding the regulation mechanism offers the potential to reprogram the HR.

**Figure 5:**
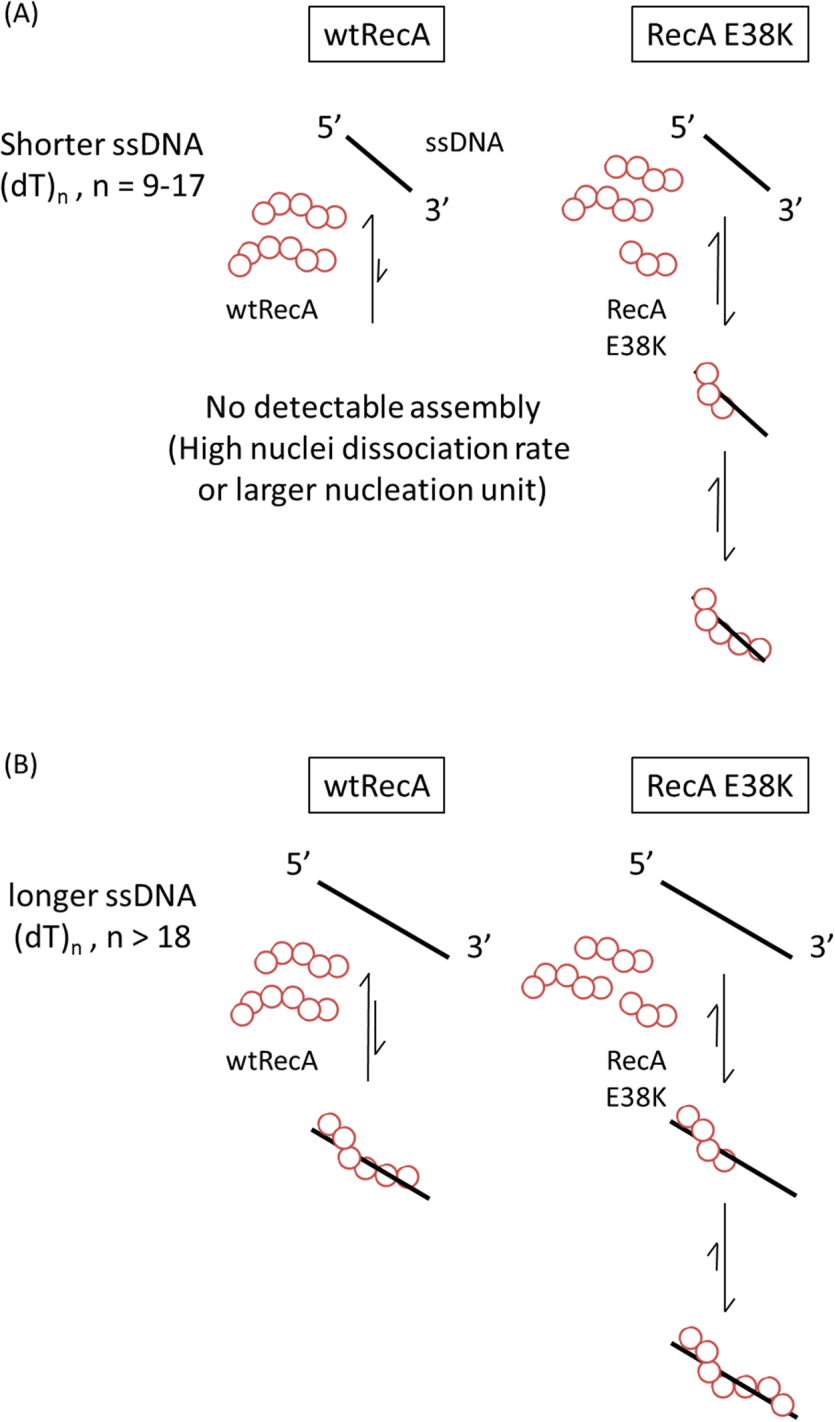
Proposed nucleoprotein filament assembly model for wtRecA and E38K. (**A**) On shorter ssDNA (9–17 nt) substrates, wtRecA cannot nucleate on ssDNA either due to a larger nucleation unit, or a high dissociation rate. While E38K is capable of forming stable clusters with much higher stability. (**B**) As ssDNA is longer (>18 nt), both wtRecA and E38K are able to form stable clusters on ssDNA.

Originally identified from UV-resistance screens, the RecA E38K protein showed a higher overall strand exchange efficiency, higher ATPase activity, and better assembly efficiency on SSB-coated ssDNA in the absence of RecFOR^10,11,19^. This suggests a single charge point mutation in RecA could alter RecA activity dramatically. Here, we study how this mutant alters the overall HR efficiency using two real-time single-molecule methods: TPM and smFRET experiments. In our TPM experiments, RecA E38K nucleated and extended at rates much faster than wtRecA on (dT)_60_ gapped DNA in the presence of ATP (Fig. 1B–D). When RecA E38K filaments were pre-formed, the RecA E38K mutant showed no stimulation of strand exchange efficiency, compared to the wtRecA (Figure S1B & S1E). Based on these two results, we propose that RecA E38K stimulates homologous DNA recombination through its enhanced assembly kinetics on ssDNA. We then used smFRET to study the initial binding and nucleation events of RecA on ssDNA with higher spatiotemporal resolution. The measured apparent nucleation frequency at various ssDNA lengths shows that RecA E38K mutant can form much more stable nuclei on shorter ssDNA, or bind ssDNA with a minimum nucleation cluster size ( < 5 monomers). Additionally, RecA E38K displays higher overall ssDNA binding affinity than wtRecA based on both the alternation events and the dwell time analyses (Figs 2D–I, 3 & Table 1). We also found that the increase in association equilibrium constants, *K*_*eq*_, of RecA E38K at longer ssDNA length results from the decrease in dissociation rate constant at long ssDNA. We proposed a model for the nucleation process of RecA E38K and wtRecA (Fig. 5). On shorter ssDNA substrates (9–17 nt), wtRecA cannot stably nucleate on ssDNA under these conditions, while RecA E38K might be capable of forming a nucleation cluster containing < 5 monomers (Fig. 5A). As ssDNA is lengthened (>18 nt), both wtRecA and RecA E38K are able to form a nucleus on ssDNA, while RecA E38K forms much more stable filaments over this size of ssDNA. Longer ssDNA substrates allow wtRecA to form nuclei with better stability, or wtRecA requires a larger nucleation cluster size of >5–6 oligomers^13,26,30^, whereas <5 RecA E38K monomers are sufficient for assembly with much-enhanced ssDNA affinity (Fig. 5B). We speculate that these two properties of RecA E38K, better nuclei stability on short ssDNA, and higher ssDNA binding affinity, largely explain the observation that a *reca730* mutant strain (RecA E38K) can survive under high UV dose condition and efficiently repair DNA^8^, and that E38K can rapidly bind on SSB-wrapped ssDNA in the absence of accessory proteins^10,23^.

It’s very surprising that a single point mutation can lead to significant enhancement of binding affinity, enzymatic activities, as well as the decrease of the nucleation unit. Surprisingly, glutamate 38 residue in RecA is positioned on the external surface of the filament and close to the monomer-monomer interface, rather far away from the DNA binding site and nucleotide grasp core domain^20^. Structural studies showed that glutamate 38 residue is in proximity with the distorted C-terminal tail in RecA, which has been reported for modulation of RecA subunit-subunit interaction^13,20,31^. It is possible that mutation on glutamate 38 position could alter the monomer-monomer interaction by interacting with the C-terminal tail to modulate the DNA binding through long-range conformational change. The mutation from an anionic glutamate residue into positively-charged lysine residue might implicate that the potential electrostatic interaction is involved in stabilizing filament structure. Several single point mutation variants of RecA have also been reported to possess enhanced recombinase activity by modulating RecA subunit-subunit interaction, such as RecA D112R, D276A, I102L and V79L. These point mutations then lead to the significant changes in DNA affinity, ATPase, and strand exchange activity^22,32,33^.

A recent biochemical study also illustrates that an efficient RecA E38K-mediated constitutive SOS response relies on RecA E38K filament formation on dsDNA. Moreover, RecA E38K hydrolyzes ATP at a faster rate than wtRecA does in the presence of dsDNA^34^. These two observations implicate the ability of RecA E38K to assemble onto dsDNA. Combined with our observation, these results suggest RecA E38K forms nucleoprotein filament faster than wtRecA does on either ssDNA or dsDNA, confirming that RecA E38K serves as an efficient recombinase and SOS response mediator.

Recent single-molecule FRET studies reported that a cooperative conformational change exists in wtRecA^35^. It was proposed that a RecA monomer in the middle of filament could continuously hydrolyze ATP without dissociation from DNA, but can bind a new ATP molecule^35^. Similar regulation strategies are found in the eukaryotic RecA-family recombinase Rad51. In both mouse and fission yeast, a Rad51 accessory protein complex, Swi5-Sfr1 has been reported to stabilize Rad51 presynaptic filaments^36–38^. Several biochemical experiments showed that Swi5-Sfr1 enhances ATPase activity of Rad51 by stimulating the dissociation of ADP hydrolysis and re-binding of a new ATP molecule to maintain the filament in an active filament during HR^38,39^. For RecA E38K, it is possible that cooperativity between adjacent RecA-RecA subunits could be stronger. We speculate that this stronger cooperativity could potentially allow RecA E38K monomers within the filament to release ADP and to efficiently re-capture a new ATP molecule to keep the filament in a stable, extended conformation. This notion is consistent with the previous studies that higher DNA affinity and greater filament stability of RecA E38K make the E38K filaments resistant to the action anti-recombinogenic helicases (e.g. UvrD) or RecA disassemblers (e.g. RecX) to dismantle nucleoprotein filaments^33,40^.

Using real-time, single-molecule TPM and FRET experiments, we showed that RecA E38K carries out homologous recombination more efficiently mainly due to a faster, stable presynaptic filament formation. We also found that efficient RecA E38K filament formation results from a greater nuclei stability and a higher ssDNA binding affinity. Considering the assembly of recombinase nucleoprotein filament is the key step in homologous recombination, stimulation at this committed step is cost-efficient. This work offers hints to engineer more efficient recombinases with potential gene editing applications.

## Materials and Methods

### DNA substrates preparation

The (dT)60 gapped DNA substrate for TPM assembly experiment contains 60 nt poly dT sandwiched by a 351 bp, 5′-digoxigenin-labeled dsDNA handle and a 5′-biotin-labeled 19 bp handle. which was prepared as previously described^23^. The ssDNA and 427/352 hybrid DNA substrates for the outgoing strand experiments were prepared as previously described21. For smFRET experiments, the surface-bound hybrid DNA substrates were prepared by annealing one 5′-Cy5 and 3′-biotin (5′-Cy5/GCCTCGCTGCCGTCGCCA/bio-3′) double-labeled oligo and one 3′-Cy3-labeled oligo with various numbers of thymidylate at 3′ overhang (5′-TGGCGACGGCaGCGAGGC (dT)n/Cy3–3′) in the buffer containing 20 mM Tris and 0.5 M NaCl at pH = 8. For bulk strand exchange assay, 40 bp homologous duplex DNA was prepared by the annealing a 5′-Cy3-tagged oligo (5′-Cy3-TAA TAC AAA ATA AGT AAA TGA ATA AAA CAG AGA AAA TAA AG-3′) with an unlabeled oligo (5′-CTT TAT TTT CTC TGT TTA TTC ATT TAC TTA TTT TGT ATT A-3′).

### Protein and buffer condition

E. coli wild-type RecA was purchased from New England Biolabs (NEB, M0249) without further purification. RecA E38K mutant was purified as previously described^11,41^, and a brief description is included in the supplemental information. All RecA reactions in tethered particle motion experiments and bulk strand exchange experiments were performed under the RecA buffer containing 25 mM Tris-HCl, 10 mM magnesium acetate, 3 mM potassium glutamate at pH 7.5^20,24^. ATP was purchased from Sigma-Aldrich.

### Bulk strand exchange assay

3 nM 80 nt ssDNA (5′-TTA TGT TCA TTT TTT ATA TCC TTT ACT TTA TTT TCT CTG TTT ATT CAT TTA CTT ATT TTG TAT TAT CCT TAT CTT ATT TA-3′) were pre-incubated with 0.3 μM RecA or RecA E38K, 1 mM DTT and 2 mM ATP in RecA buffer at 37 °C for 15 min to form nucleoprotein filament. 40 bp Cy3-labeled homologous dsDNA was prepared by annealing an ordinary primer with a 5′-Cy3-tagged primer. RecA filaments were then challenged with 2 nM 40 bp Cy3-labeled homologous dsDNA and reacted for the indicated times (0, 1, 2, 4, 7, 10, 20 min) at 37 °C. Strand exchange reactions at given time were stopped by adding 0.5% SDS and 6 μg Proteinase K into reaction mixture and incubating for 10 min at 37 °C. The products were resolved under 8% PAGE in 1xTAE, 125 Volt for 55 min at room temperature. Cy3 fluorescence was imaged under 532 nm laser excitation and images were quantified using GE Typhoon Trio.

### Single-molecule tethered particle motion (TPM) assembly experiment and data analysis

Streptavidin beads were prepared as previously described^42^. In filament assembly experiments, 4 nM (dT)_60_ gapped ssDNA substrates were immobilized on the anti-digoxigenin-coated coverslip and incubated for 30 min followed by buffer washing to remove unbound DNA. 220 nm streptavidin-tagged polystyrene beads were then attached to the DNA substrates for imaging. 1 μM RecA or RecA E38K with 1 mM DTT and 2 mM ATP in RecA buffer was pre-incubated at 37 °C for 10 min. After cooling down to the room temperature, the RecA mixture was flowed into the reaction chamber containing bead-labeled (dT)60 gapped DNA substrates. We utilized an inverted optical microscope (IX-71, Olympus) using a differential interference contrast (DIC) imaging method to visualize tethers and measure bead Brownian motion (BM). Images of assembly experiments were acquired at 33 ms resolution using a Newvicon camera (Dage-MTI) and were analyzed using software written in Labview. The amplitude of tether Brownian motion is defined by the standard deviation of the bead centroid positions of 20 images using a sliding window. For each independent assembly experiments, images were first recorded for around 30 seconds (~1000 image frames) before the introduction of RecA mixture and then for about 10 minutes (~20000 image frames) after the addition of RecA mixture.

### Single-molecule TPM outgoing strand experiment and data analysis

2 nM of 5′-digoxigenin and 5′-biotin double labeled 427/352 hybrid DNA was specifically then anchored on the anti-digoxigenin-covered glass surface. Streptavidin beads were further introduced into reaction chambers for visualization. Beads were labeled on the strand which is homologous to invading E38K-bound ssDNA. RecA E38K nucleoprotein filaments were prepared by mixing 4 nM unlabeled 427 nt complementary ssDNA with 2 μM RecA E38K, 2 mM ATP and ATP regeneration system in RecA buffer containing 1 mM DTT at 37 °C for 10 min. Outgoing strand reactions were initiated by challenging the bead-labeled, surface-anchored 427/352 hybrid DNA with RecA filaments. We followed the reaction for 30 minutes. Only the reactions involving tethers with correct initial BM values (29.6 ± 5.18 nm for the 427/352 hybrid DNA) were taken into strand exchange efficiency measurement. Under our single-molecule TPM condition, the surface tether density is around 50–100 DNA tethers per field by counting the numbers of bead-probed 427/352 hybrid DNA and gapped DNA at the beginning (t = 0) of experiments.

### Single-molecule fluorescence resonance energy transfer (smFRET) experiment and data analysis

Glass slides and coverslips were first cleaned by sonicating slides and coverslips in 2 M KOH for 15–20 min, in 99% ethanol for 15–20 min and 1 M KOH for 15–20 min sequentially. After sonication, coverslips and slides were washed with ddH_2_O and cleaned by using plasma cleaner. Glass slides were then functionalized in a solution of 3-aminopropyltrimethoxysilane (Sigma-Aldrich) in acidic methanol in the dark for 20 min at room temperature. After functionalization, slides, and coverslips were rinsed with methanol and ddH2O alternatively and dried by blowing N2. Slides and coverslips were PEGylated in a mixture of 0.16% w/v biotin-PEG (MW 5000, Laysan Bio, Inc.) and 33% w/v PEG (MW 5000, Laysan Bio, Inc.) in 0.1M sodium bicarbonate The slides and coverslips were incubated with PEG mixture solution for 4 hours in the dark. As PEGylation was done, slides and coverslips were rinsed with ddH_2_O alternatively and dried with N2. Reaction chambers were prepared by adhering double-sided tape strips (3M, Inc.) on a slide followed by attaching a coverslip on double-sided tape strips on the slide. To perform smFRET experiments, reaction chambers were coated with 20 μg/mL streptavidin and incubated for 5 min. Excess streptavidin was washed away with the buffer containing 20 mM Tris and 50 mM NaCl. 15 pM of 3′-biotinylated dye-labeled hybrid DNA was then anchored on the surface for 5 min. After 5 min incubation, free DNA was washed with RecA imaging buffer containing 1 mM Trolox (Sigma-Aldrich), 2.6 mM protocatechuic acid (PCA, Sigma-Aldrich), 0.21 units/mL protocatechuate 3,4-dioxygenase (PCD, Sigma-Aldrich), 20 mM Tris, 10 mM magnesium acetate and 3 mM potassium glutamate at pH 7.8. The RecA reaction was initiated by flowing a mixture of the solution including 1 μM RecA or RecA E38K and 2 mM ATP in RecA imaging buffer into reaction chambers. Under our conditions, 90% of fluorophores in hybrid DNA can survive for more than 200 sec.

We used objective-type total internal reflection fluorescence microscope (TIRFM, Olympus IX2) and 532 nm laser as excitation light source to collect all smFRET data. Fluorescence intensity signals of both Cy3 and Cy5 were acquired by EMCCD (ProEM 512B, Princeton Instrument) at 50 ms resolution after emission beams pass through the dual view system. Cy3 and Cy5 fluorescence emission movies were recorded using a software program written in Labview 8.6. Fluorescence emission movie files were then used for colocalization analysis using a mapping software program written in IDL to find co-localized spot pairs in movie files. After mapping, fluorescence intensity time trace of each single DNA molecule can be obtained using Matlab program. FRET values, nucleation frequency and assembly/ disassembly rate constants of each molecule were analyzed using Hidden Markov model^26^.

## Electronic supplementary material

Supplemental Information
