# Supplementary material for: Stable Nuclei of Nucleoprotein Filament and High ssDNA Binding Affinity Contribute to Enhanced RecA E38K Recombinase Activity
Source: Sci Rep. 2017 Nov 2;7:14964. doi: 10.1038/s41598-017-15088-z (PMC5668366; doi:10.1038/s41598-017-15088-z)
Supplement: Supplementary file 1 — Supplemental Information [file 41598_2017_15088_MOESM1_ESM.pdf]

Supplemental Information for

Stable Nuclei of Nucleoprotein Filament and High ssDNA Binding Affinity Contribute to Enhanced RecA E38K Recombinase Activity

Chih-Hao Lu[#], Ting-Tzu Chang[#], Chia-Chuan Cho, Hui-Cin Lin, and Hung-Wen Li[*]

Department of Chemistry, National Taiwan University, Taiwan

[*]To whom correspondence should be addressed: Hung-Wen Li, E-mail hwli@ntu.edu.tw

[#]C.H.L. and T.T.C. contributed equally to this work.

RecA E38K Purification

RecA E38K protein was purified as previously described[1,2]. Cell paste was thawed and re-suspended in a solution of 25% (w/v) sucrose and 250 mM Tris (pH 7.5). Cells were lysed by a 60-min incubation with a solution of lysozyme in 250 mM Tris (pH 7.5), followed by the addition of 50 ml of 25 mM EDTA, sonication, and centrifugation. The lysate was precipitated with 5% (w/v) polyethyleneimine (pH 7.5) and centrifuged. The pellet was washed and extracted with R buffer (20 mM Tris-HCl, 80% cation, pH 7.5) plus ammonium sulfate. After that, the protein solution was then precipitated by adding ammonium sulfate. The resulting pellet was washed with R buffer (with 2.1 M ammonium sulfate), re-suspended in R buffer (with 50 mM KCl), and then dialyzed. The protein solution was then loaded onto a DEAE-Sepharose column and washed with R buffer (with 50 mM KCl). Peak fractions of flow-through were resolved by SDS-PAGE, pooled, and dialyzed. The protein was loaded onto a PBE 94 column with R buffer, and the column was developed with a linear gradient of KCl. The RecA E38K mutant was eluted, dialyzed extensively in R buffer and concentrated using Centricon (Amicon).

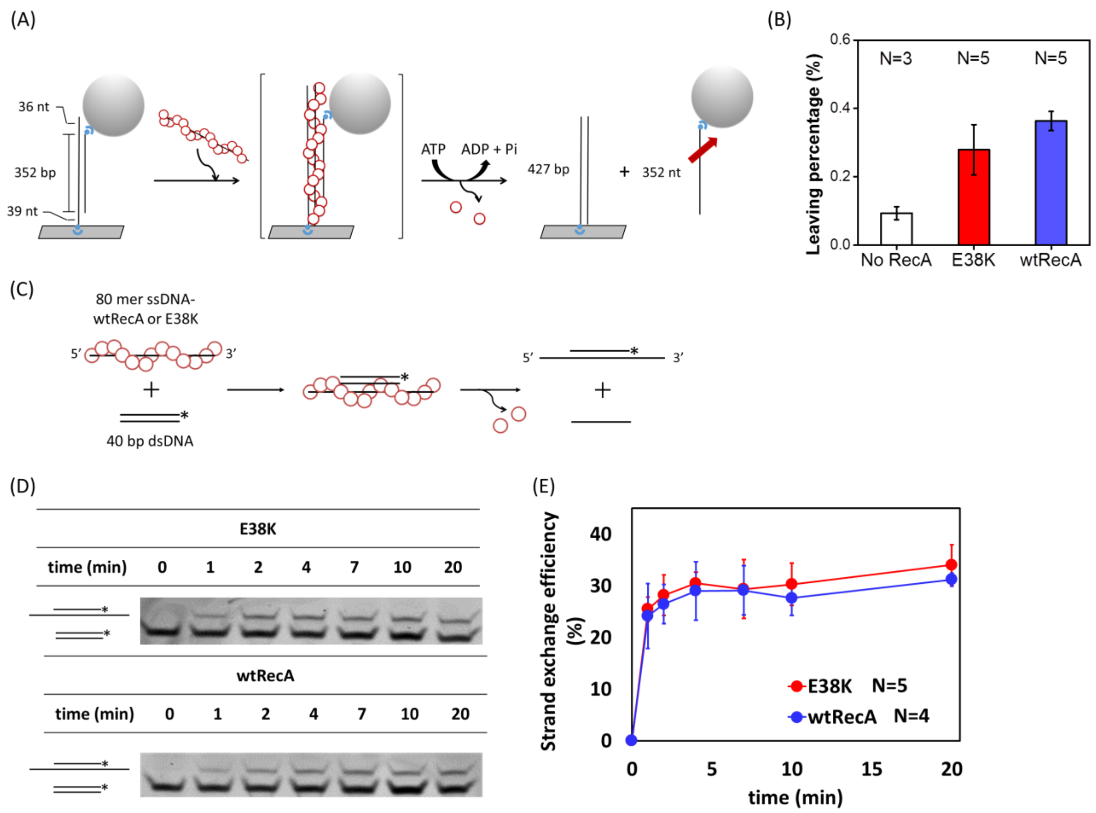

Figure S1. Comparison of strand exchange efficiency between RecA E38K and wild-type RecA. (A) Schematic illustration of the TPM outgoing strand experiment. Bead-labeled, surface-bound 427/352 hybrid DNA was challenged by pre-incubated, RecA-coated ssDNA. At the completion of the strand exchange, fully duplex 427 bp DNA is formed and bead-labeled 352 nt strand is displaced. Tether leaving percentage thus reflects the strand exchange efficiency. (B) No significant difference in strand exchange efficiency was seen between wtRecA and RecA E38K measured at the 35 minutes after protein addition. *N* indicates the number of independent experiments. (C) Schematic illustration of gel-based strand exchange experiments. 5′-Cy3-labeled 40 bp dsDNA was reacted with pre-incubated, RecA-coated 80 mer ssDNA. Sequence of the center region of this 80 mer ssDNA is complementary to the 40 bp dsDNA. Strand exchange reactions were initiated by mixing DNA substrates and ATP-containing buffer. The reactions were stopped at specified time points by adding SDS and proteinase K. Strand exchange product was a Cy3-tagged 40/80 hybrid DNA. (D) Polyacrylamide gel images of time courses of E38K (upper) and wtRecA (lower) strand exchange. (E) Strand exchange efficiency is plotted as a function of time. No significant difference in strand exchange kinetics was found between wtRecA and RecA E38K. Native PAGE experiments were repeated for at least three times. *N* indicates the number of independent experiments. Error bar is one standard error of the mean.

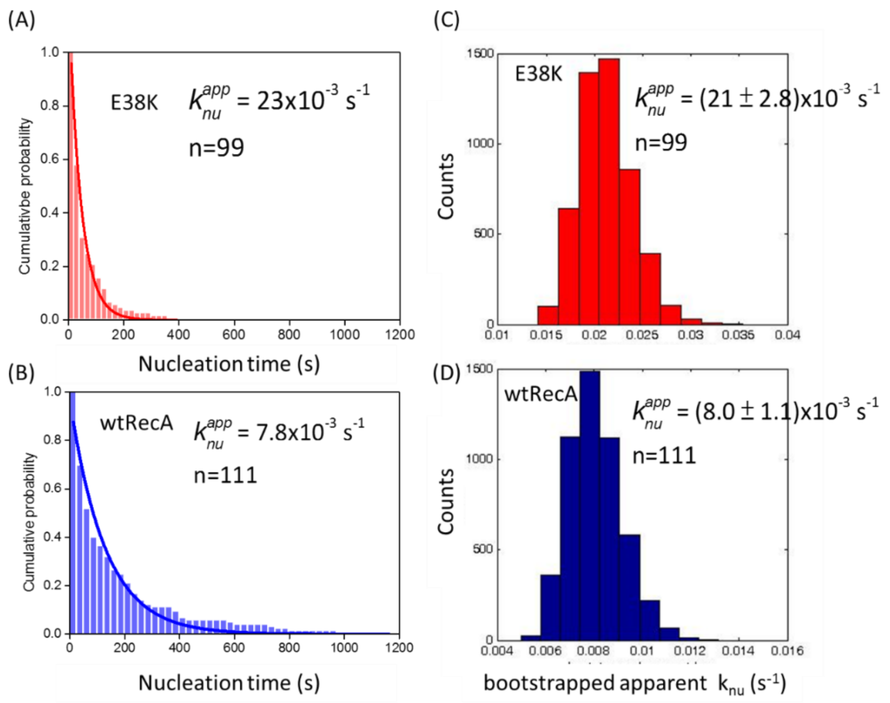

Figure S2. Histograms of apparent k_{nu} (C) & (D) originated from the fitting of 5,000 bootstrapped samples of the data in (A) & (B). Apparent k_{nu} in (A) & (B) were acquired by fitting nucleation time histograms with single-exponential decay. In (C) & (D), the mean of apparent k_{nu} is calculated based on Maximum likelihood estimation (MLE)[3]; The standard deviation is the estimated standard error of apparent k_{nu} of two proteins. These two measurements give similar results. *n* indicates the number of tethers compiled from more than 3 independent experiments.

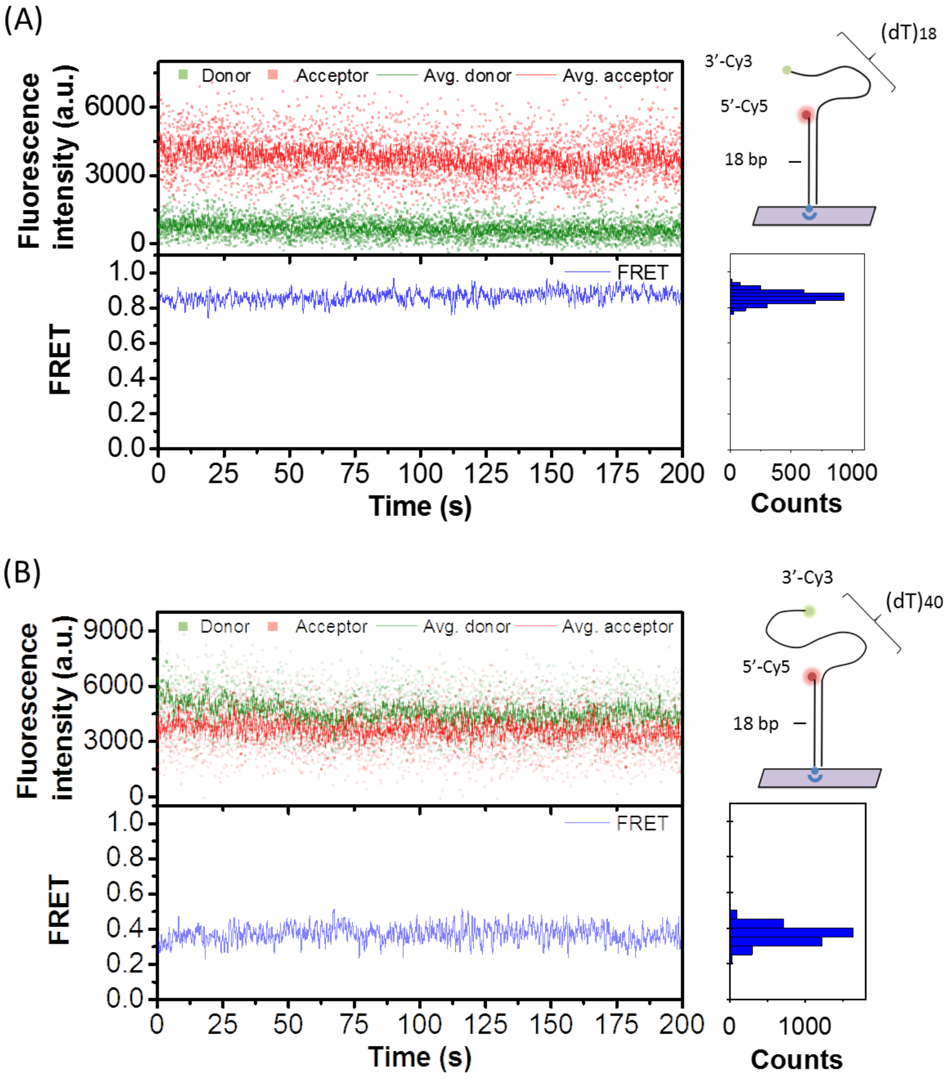

Figure S3. Representative FRET time trace of (A) bare $(dT)_{18}$ and (B) bare $(dT)_{40}$ DNA substrate. In the absence of proteins, DNA molecules exist only in higher FRET state owing to the flexibility of ssDNA. At shorter $(dT)_n$ lengths, the FRET values are high (~ 0.85 for $(dT)_{18}$), while at longer $(dT)_n$ lengths, the FRET values are a bit lower (~ 0.35 for $(dT)_{40}$) due to the increasing ssDNA length.

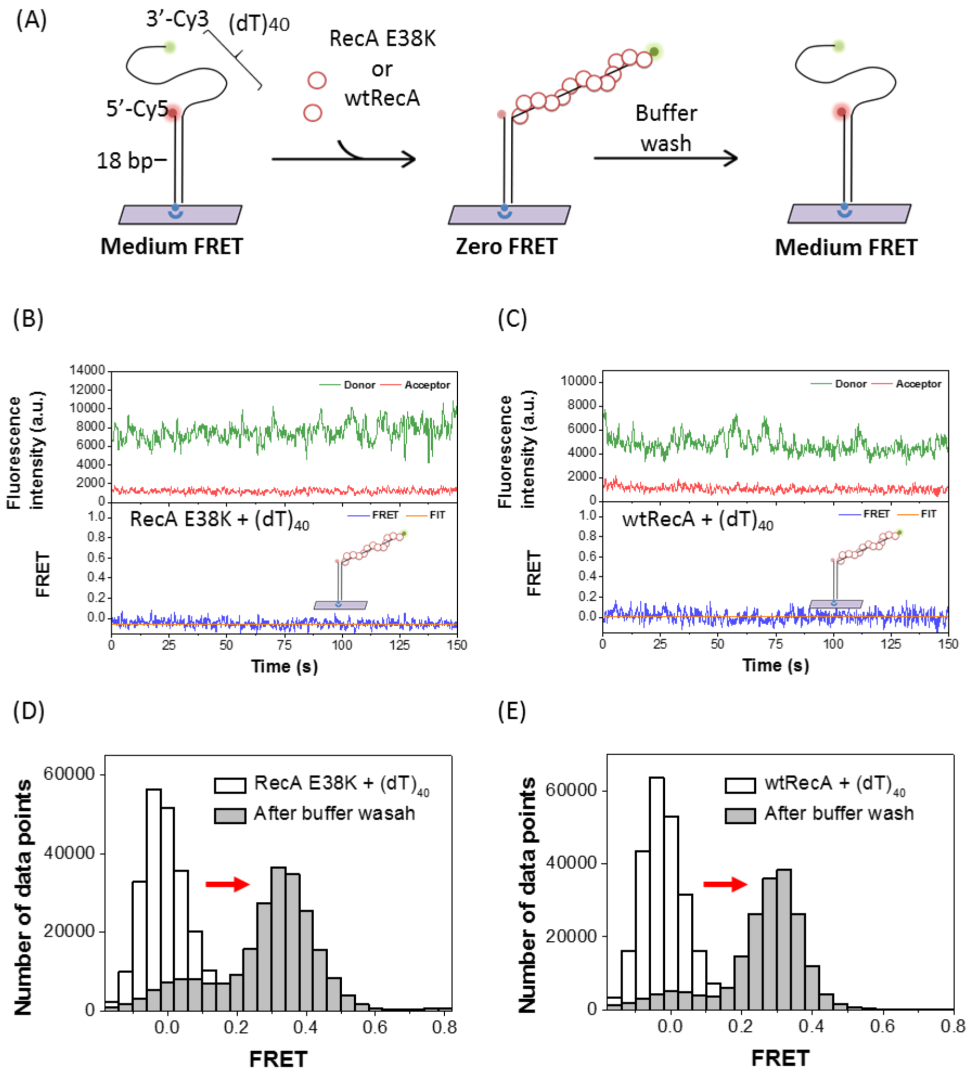

Figure S4. FRET experiments can monitor the disassembly of RecA E38K and wtRecA from $(dT)_{40}$ substrate after extensive buffer washing. (A) Using a longer $(dT)_{40}$ substrate (medium FRET), both RecA E38K and wtRecA can stably stay bound to ssDNA (low FRET). (B-C) FRET time traces of RecA E38K or wtRecA assembly on $(dT)_{40}$ ssDNA overhangs. The assembly is fast and has taken place before the recording. Each time-traces were fitted by the hidden Markov model (orange line). FRET efficiency stays in the very low FRET state since both RecA E38K and wtRecA remain stably bound when the ssDNA length is sufficiently long. To confirm that the low FRET state is not resulted from the Cy5 photobleaching, we initiated RecA disassembly by extensive buffer wash. Both (D) E38K and (E) wtRecA dissociated from DNA after buffer wash, where FRET returned to ~ 0.3, corresponding to the $(dT)_{40}$ DNA-only state. Smaller peaks at E ~ 0 in both (B) and (C) indicated that small fraction of DNA molecules remained stably bound by recombinase after the buffer wash.

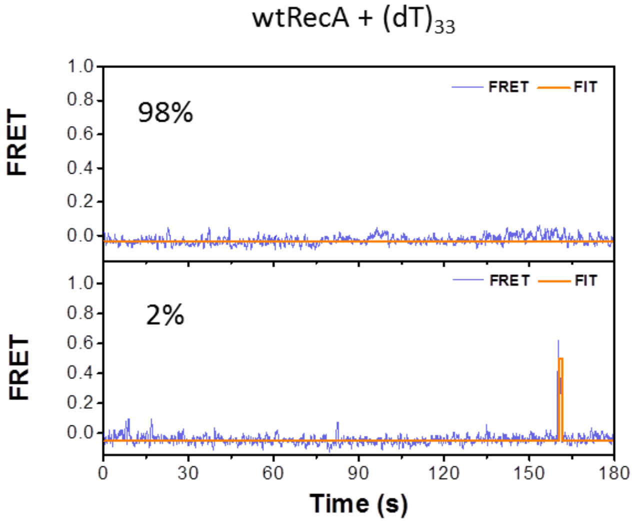

Figure S5. Two types of FRET time traces of wtRecA assembling on $(dT)_{33}$ ssDNA substrate. ~98% of wtRecA are stably bound on $(dT)_{33}$ without dissociating from ssDNA (upper panel). Even though we discovered that ~2% of wtRecA could dissociate from the $(dT)_{33}$ substrate, the rebinding is fast (very transient high FRET state).

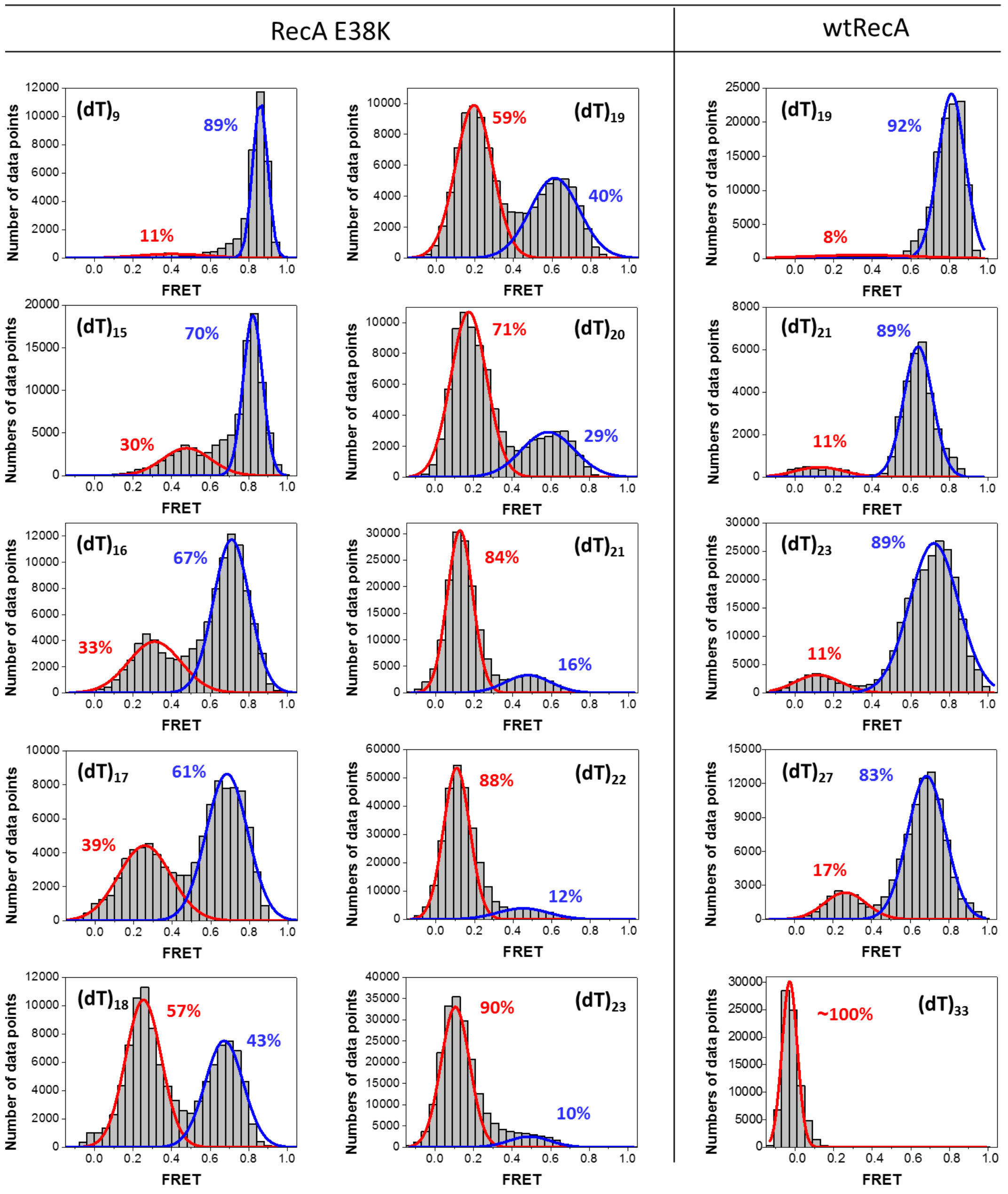

Figure S6. The FRET histograms of DNA molecules of various lengths in the presence of RecA E38K (Left panel) or wtRecA (Right panel). The two peak centers represent the protein-bound state (FRET ~ 0.1-0.3, red peaks) and protein-free state (FRET ~ 0.6-0.9, blue peaks), respectively.

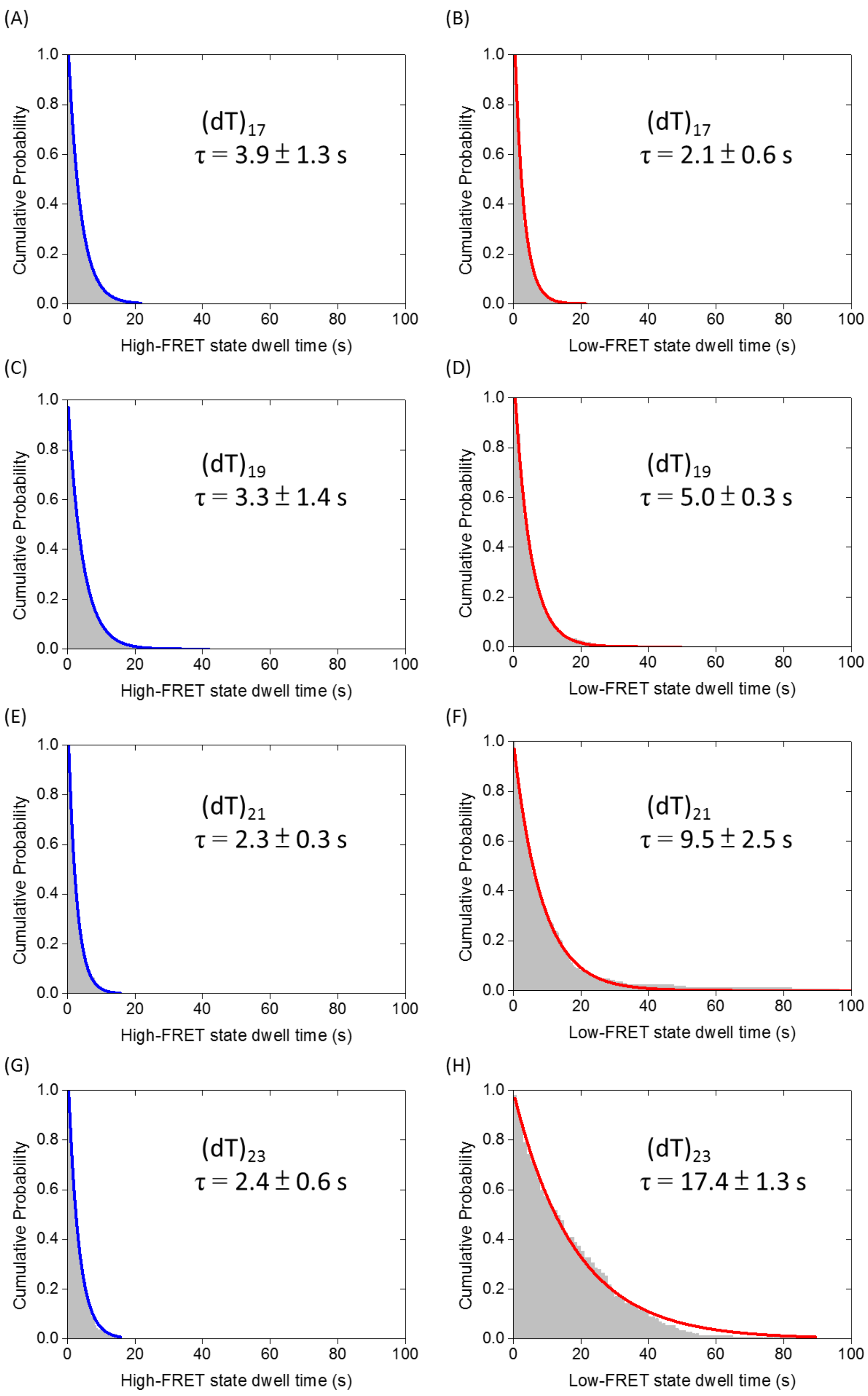

Figure S7. Histograms of high-FRET and low-FRET dwell times of RecA E38K on various ssDNA substrates. Mean and error bar shown in each histogram were compelled from at least three independent experiments. The bin sizes of the histograms are 0.5 seconds for high-FRET state dwell time (A, C, E, and G) and 1.0 second for low-FRET state dwell time (B, D, F, and H).

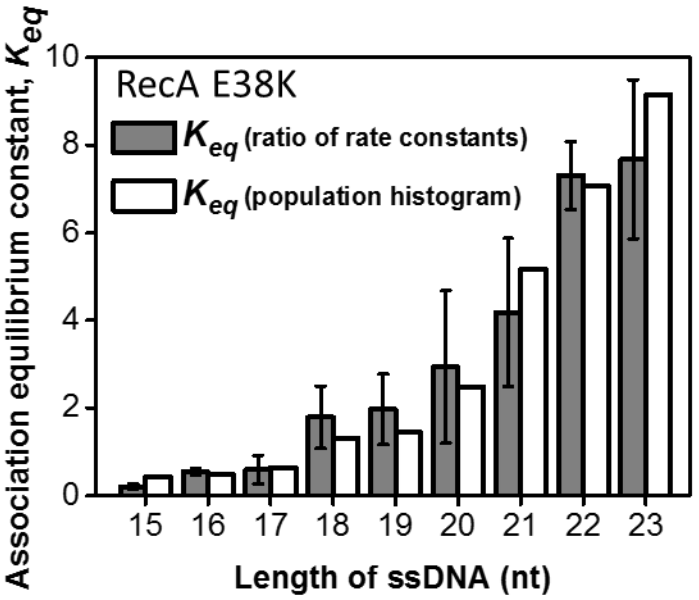

Figure S8. Association equilibrium constants of RecA E38K assembling on various ssDNA substrates obtained from the population histograms and from the ratio of rate constants are similar. K_{eq} (ratio of rate constants) is the ratio of the apparent nucleation cluster formation rate constant to the dissociation rate constant, determined from the Hidden Markov Model fitting (Figure 2D-2I). K_{eq} (population histogram) is the population ratio of the RecA E38K-bound DNA molecules to the amount of bare DNA molecules, based on the BM histograms from Figure S6. Two different methods return with similar equilibrium constants, confirming the reliability of the data analysis and the fitting algorithm.

Length of ssDNA (nt)	Dwell time of high-FRET state (s)	k_f^{App} (s^{-1})	Dwell time of low-FRET state (s)	k_d (s^{-1})	Association equilibrium constant, K_{eq}^1	Association equilibrium constant, K_{eq}^2
15	4.4 ± 0.4	0.23 ± 0.02	0.89 ± 0.2	1.16 ± 0.26	0.21 ± 0.06	0.42
16	3.1 ± 0.3	0.32 ± 0.03	1.7 ± 0.4	0.60 ± 0.13	0.54 ± 0.07	0.48
17	3.9 ± 1.3	0.27 ± 0.09	2.1 ± 0.6	0.50 ± 0.14	0.59 ± 0.33	0.65
18	2.4 ± 0.1	0.41 ± 0.01	4.4 ± 1.7	0.25 ± 0.09	1.79 ± 0.71	1.30
19	3.3 ± 1.4	0.39 ± 0.15	5.0 ± 0.3	0.20 ± 0.01	1.97 ± 0.80	1.44
20	3.4 ± 1.2	0.33 ± 0.13	8.5 ± 2.0	0.12 ± 0.03	2.93 ± 1.74	2.49
21	2.3 ± 0.3	0.43 ± 0.06	9.5 ± 2.5	0.11 ± 0.03	4.18 ± 1.69	5.16
22	2.4 ± 0.1	0.42 ± 0.03	17.4 ± 0.6	0.06 ± 0.00	7.31 ± 0.77	7.08
23	2.4 ± 0.6	0.44 ± 0.11	17.4 ± 1.3	0.06 ± 0.00	7.68 ± 1.82	9.14

Table S1. The mean dwell time of two FRET states, corresponding kinetic parameters and association equilibrium constants of RecA E38K on the various length of ssDNA. Each average was compiled from at least 3 independent experiments. Error bar is the standard deviation.

[1] K_{eq} (ratio of rate constants), the ratio of the apparent nucleation cluster formation rate constant to the dissociation rate constant.

[2] K_{eq} (population histogram), the ratio of the amount of RecA E38K-bound DNA molecules to the amount of bare DNA molecules, based on the BM histograms from Figure S6.

References

1 Eggler, A. L., Lusetti, S. L. & Cox, M. M. The C terminus of the Escherichia coli RecA protein modulates the DNA binding competition with single-stranded DNA-binding protein. *J Biol Chem* **278**, 16389-16396 (2003).

2 Lusetti, S. L. *et al.* C-terminal deletions of the Escherichia coli RecA protein - Characterization of in vivo and in vitro effects. *J Biol Chem* **278**, 16372-16380 (2003).

3 Friedman, L. J. & Gelles, J. Multi-wavelength single-molecule fluorescence analysis of transcription mechanisms. *Methods* **86**, 27-36 (2015).
